# Identification of a novel mutation in the *PRCD* gene causing autosomal recessive retinitis pigmentosa in a Turkish family

**Published:** 2013-06-13

**Authors:** Johanna Pach, Susanne Kohl, Florian Gekeler, Ditta Zobor

**Affiliations:** 1 Centre for Ophthalmology, University of Tübingen; 2 Institute for Ophthalmic Research, Molecular Genetics Laboratory, University of Tübingen; 3 Institute for Ophthalmic Research, University of Tübingen, Germany

## Abstract

**Purpose:**

Progressive rod-cone degeneration (*PRCD*) is a canine form of autosomal recessive photoreceptor degeneration and serves as an animal model for human retinitis pigmentosa (RP). To date, only two RP-causing mutations of the *PRCD* gene have been reported in humans. We found a novel mutation in *PRCD* (c.52C>T, p.R18X) in three siblings affected by RP and present detailed morphologic and functional parameters.

**Methods:**

A complete ophthalmological examination was performed including psychophysical tests (best-corrected visual acuity, Lanthony Panel D-15 color vision test, and visual field) and electrophysiology (ganzfeld and multifocal electroretinogram). Additionally, color and infrared fundus photography, autofluorescence, and spectral domain optical coherence tomography recordings were performed. Genomic DNA of the three affected individuals was analyzed with high-throughput sequencing for all RP-related genes in a diagnostic set-up.

**Results:**

We identified a novel homozygous mutation in *PRCD* (c.52C>T, p.R18X) with diagnostic high-throughput panel sequencing. All three patients showed an advanced stage of retinitis pigmentosa with reduced visual acuity (mean: 20/80), small residual visual fields (mean for target III4e: 1134.35 deg^2^), and non-detectable electrophysiological responses. Myopia, posterior subcapsular cataract, bone spicule-like pigmentation, and attenuated arterioles were typical findings. Interestingly, bull’s eye maculopathy due to patchy retinal pigment epithelium atrophy was also present in all patients. The mean central retinal thickness observed in optical coherence tomography was 148 µm.

**Conclusions:**

The identification of a third mutation in *PRCD* confirms its role in the pathogenesis of RP. Clinical findings were in line with the morphological changes observed in previous studies. Bull’s eye maculopathy seems to be a hallmark of RP due to mutations in the *PRCD* gene.

## Introduction

Retinitis pigmentosa (RP, OMIM 268000) is a clinically and genetically heterogeneous group of hereditary retinal disorders, and one of the most common types of retinal degeneration with a prevalence of 1:4,000 [1]. More than 40 genes have been associated with RP so far, whose defects cause a progressive loss of rod photoreceptor function, followed by cone photoreceptor dysfunction. Typical symptoms are night blindness, loss of peripheral vision, and later central vision in advanced stages of disease, often leading to complete blindness. Non-syndromic RP can show all types of inheritance: autosomal dominant, autosomal recessive, or X-linked; even mitochondrial and digenic inheritance has been reported [2]. Among the genes already identified, at least 23 are associated with an autosomal recessive type of inheritance (RetNet; The Retinal Information Network. 2012).

Progressive rod-cone degeneration (*PRCD*) is a canine, late-onset form of retinal dystrophy that has been mapped to the canine chromosome 9 [3,4]. The *PRCD* (OMIM 610598; homolog of dog PRCD) gene in humans is located on chromosome 17q25.1 and encodes a small protein of 54 amino acids, but the molecular etiology and function are unknown. To date, only two RP-causing mutations of the *PRCD* gene have been reported in humans: a homozygous missense mutation (c.5G>A p.C2Y) in a single patient from Bangladesh and a homozygous nonsense mutation (c.64C>T p.R22X) in patients with RP in a small Arab village [3,5]. We present a family with three affected siblings carrying a novel RP-causing mutation in the *PRCD* gene.

## Methods

### Clinical assessment

**Figure 1:**
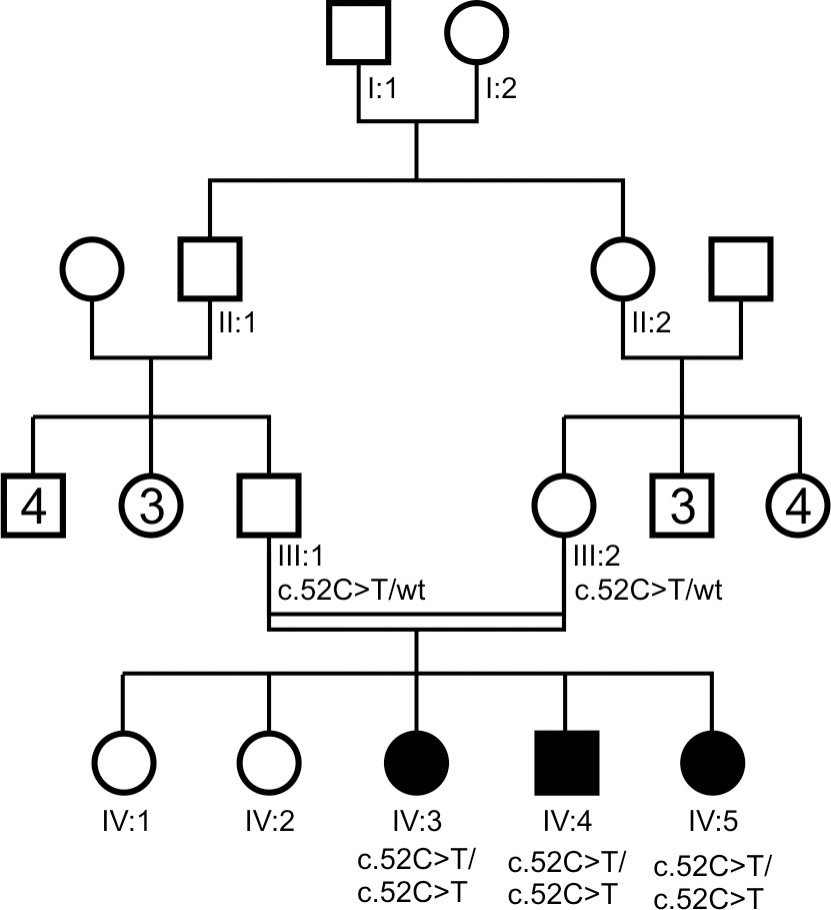
Pedigree and genotypes. All three affected siblings are homozygous for the progressive rod-cone degeneration (*PRCD*) mutation c.52C>T p.R18X, while the parents (who are first-degree cousins) are heterozygous carriers.

Three affected siblings (two women and one man) of a family of Turkish origin were examined in the Centre for Ophthalmology in Tübingen, Germany (Figure 1). The affected patients were otherwise healthy. Informed consent was obtained from all patients, and the examinations were performed respecting the Code of Ethics of the World Medical Association (Declaration of Helsinki). Approval of the local ethics committee was obtained. A complete ophthalmological examination was performed including psychophysical tests (best-corrected visual acuity, Lanthony Panel D-15 color vision test, and visual field) and electrophysiology (Ganzfeld and multifocal electroretinogram [ERG]).

Visual field tests were performed using an Octopus 900 perimeter (Haag-Streit International, Köniz, Switzerland). Semiautomated kinetic perimetry using Goldmann stimulus V4e, III4e, and an additional dimmer stimulus, if possible, within the 90° visual field was performed in all patients.

All electrophysiological recordings were performed according to International Society for Clinical Electrophysiology of Vision Standards (ISCEV) [6,7]. Ganzfeld ERGs were performed using Dawson-Trick-Litzkow (DTL) fiber electrodes with an Espion E^2^ or E^3^ (Diagnosys LLC, Cambridge, UK) recording device coupled with a ColorDome (Diagnosys LLC) as light source. Multifocal ERG was performed with an Espion System (Diagnosys LLC). The stimulus, consisting of 61 scaled hexagonal elements, covered a central visual field of 60° × 55°. Responses were amplified (200,000X), bandpass-filtered (10–100 Hz), and analyzed according to ring averages. The same DTL electrodes as those for the Ganzfeld recordings were used. Detailed morphological examination, color and infrared fundus photography, autofluorescence, and spectral domain optical coherence tomography (OCT; Heidelberg Engineering GmbH, Heidelberg, Germany) were performed in all patients.

### Genetic analysis

Genomic DNA was extracted according to standard procedures. Peripheral blood was drawn in EDTA-blood tubes and stored at 4 °C for short-term and -20 °C for long-time storage. DNA was extracted with the automated chemagic MSM I system applying the chemagic DNA blood kit out of 5ml EDTA-blood (chemagen, Perkin Elmer, Baesweiler, Germany). Genetic testing for all genes associated with autosomal recessive retinitis pigmentosa was conducted in a diagnostic set-up (CeGaT GmbH, Tübingen, Germany) applying next-generation sequencing on a SOLiD 5500×l platform. Briefly, sequence reads were analyzed using LifeScope software package (Life Technologies GmbH, Darmstadt, Germany) and mapped to the human reference genome (GRCh37/hg19). Variant calling was generated by the LifeScope software package and annotated using the Ensembl database, Single Nucleotide Polymorphism database (dbSNP), and CeGaT in-house variant databases. Variants with a global minor allele frequency of below 5% based on the dbSNP and Exome Variant Server were further evaluated. All mutations were validated with conventional Sanger sequencing. Underrepresented regions (>10 reads per base) were examined with Sanger sequencing (detailed description of the genetic test is described in detail by Glöckle et al [8]). Segregation analysis in the three affected siblings and parents was performed with Sanger sequencing.

## Results

### Clinical findings

In a family of five siblings (four women, one man), three were affected by RP (two women, one man). All patients noticed difficulty with night vision approximately at the age of 8 to 10 years. Their parents were first-degree cousins and had normal vision. There were no other cases of RP known in the family.

**Figure 2:**
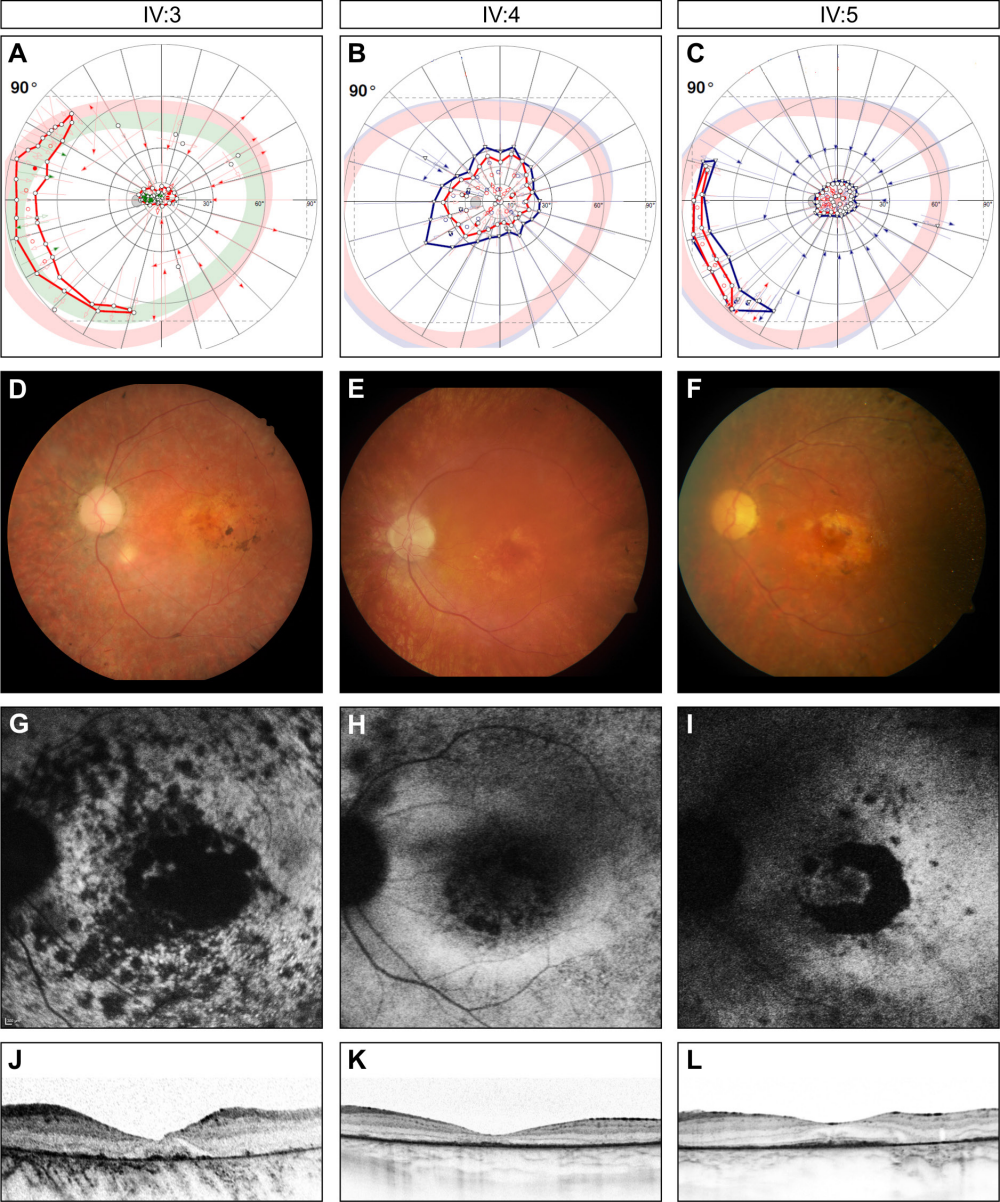
Clinical findings of the three siblings carrying a homozygous mutation in the progressive rod-cone degeneration (*PRCD*) gene. **A**–**C**: Perimetry shows visual field constriction in every patient with residual peripheral islands in the two female patients (target color coding: green: I4e, red: III4e, blue: V4e). **D**–**F**: Fundus photography shows bone-spicule-like pigmentation, attenuated arterioles and bull´s eye maculopathy (BEM) due to a patchy RPE atrophy. **G**–**I**: FAF shows sharply demarcated, smaller and larger, partly confluent areas of reduced autofluorescence. **J**–**L**: Optical coherence tomography (OCT) imaging shows a decrease in retinal thickness with shortening of the photoreceptor outer segments, reduction in the outer nuclear layer, and RPE atrophy. (Findings are shown on the left for patient IV:3, in the middle for patient IV:4, and on the right for patient IV:5.)

All three patients showed advanced signs of disease with reduced central visual acuity, color confusion, and severe constriction of the visual field (Table 1, Figure 2A-C). Nystagmus has never been reported, and pupillary reactions were normal in all cases. Electrophysiological examinations did not yield reproducible responses in any patient. Anterior segment examinations revealed subcapsular posterior cataract in each case. Typical findings on funduscopy were bone spicule-like pigmentation, attenuated arterioles, and optic disc pallor, all characteristic of RP (Figure 2D–F). Interestingly, bull’s eye maculopathy (BEM) due to patchy or geographic atrophy of the retinal pigment epithelium (RPE) was observed in every patient. This could nicely be seen in the autofluorescence images as sharply demarcated, smaller and larger, partly confluent areas of reduced autofluorescence (Figure 2G-I). OCT imaging showed an overall decrease in retinal thickness with shortening of the photoreceptor outer segments, reduction in the outer nuclear layer, and RPE atrophy (Table 1, Figure 2J-L). The inner retinal layers were less affected.

**Table 1:** Morphological and functional findings

Gender	IV:3	IV:4	IV:5
	female	male	female
Age at examination (years)	26	24	22
BCVA/ Refraction	RE: 20/50 (−1,75 sph −0,25 cyl 138°)	RE: 20/80 (−2,0 sph −0,75 cyl 42°)	RE: 20/40 (−3,75 sph −1,0 cyl 179°)
	LE: 20/630 (−2,5 sph −1,0 cyl 22°)	LE: 20/250 (−1,75 sph −1,0 cyl 175°)	LE: 20/50 (−4,5 sph −1,0 cyl 157°)
Kinetic Visual Field (target III4e)	visual field constriction: III4e: remaining island in the center of 162.0 deg^2^ (RE), 165.5 deg^2^ (LE), small remaining island in the temporal periphery of 1012.7 deg^2^ (RE), 1401.9 deg^2^ (LE)	visual field constriction: III4e: remaining island in the center of 1203.5 deg^2^ (RE), 1528.0 deg^2^ (LE)	visual field constriction: III4e: remaining island in the center of 269.2 deg^2^ (RE), 265.8 deg^2^ (LE), small remaining island in the temporal periphery of 392.4 deg^2^ (RE), 405.1 deg^2^ (LE)
Electrophysiology (Ganzfeld and mfERG)	non-detectable	non-detectable	non-detectable
Funduscopic findings	bone-spicule-like pigmentation;	bone-spicule-like pigmentation;	bone-spicule-like pigmentation;
	attenuated arterioles;	attenuated arterioles;	attenuated arterioles;
	bull’s eye maculopathy;	patchy RPE-atrophy in the macula	bull’s eye maculopathy
	optic disc pallor		
Additional findings	posterior subcapsular cataract	posterior subcapsular cataract	posterior subcapsular cataract
Foveal thickness (OCT)	RE: 154 µm	RE: 104 µm	RE: 189 µm
	LE: 126 µm	LE: 115 µm	LE: 202 µm

BCVA: best corrected visual acuity, RE: right eye, LE: left eye, sph: sphere, cyl: cylinder, non-detectable RPE: retinal pigment epithelium, OCT: spectral domain optical coherence tomography

### Genetic findings

**Figure 3:**
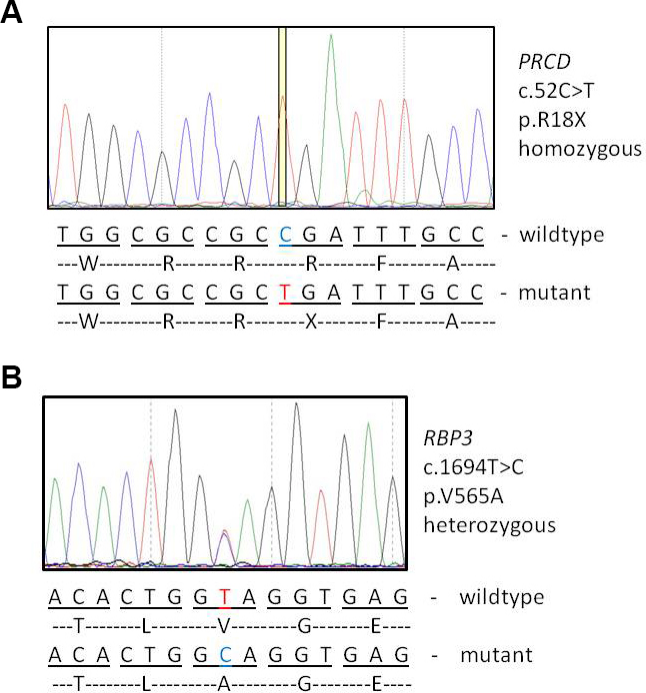
Genetic findings. **A**: Sequence electropherogram displaying the homozygous progressive rod-cone degeneration mutation c.52C>T p.R18X. **B**: Sequence electropherogram displaying the heterozygous RBP3 mutation c.1694T>C p.V565A. Beneath the electropherogram, the wild-type and mutant nucleotide-encoded amino acid sequences are shown.

Diagnostic testing via next-generation sequencing and confirmation with Sanger sequencing in the index patient (IV:3) identified an apparent homozygous nonsense mutation c.52C>T p.R18X in exon 1 of the *PRCD* gene (Figure 3A), as well as a heterozygous sequence variant c.1694T>C p.V565A in exon 1 of the interphotoreceptor retinoid-binding protein (*RBP3*) gene (Figure 3B). Segregation analysis in the two affected siblings and parents via Sanger sequencing showed that both siblings were also homozygous for the *PRCD* mutation and heterozygous for the *RBP3* variant. Both parents were heterozygous for the *PRCD* mutant allele.

## Discussion

PRCD is a canine form of autosomal recessive photoreceptor degeneration that serves as an animal model for RP in humans. The disease-relevant gene was mapped to the centromeric region of the canine chromosome 9, a region orthologous to the telomeric end of the human chromosome 17 [9,10]. Zangerl and coworkers showed that *PRCD* encodes for a small 54 amino acid protein in the dog and human, and a 53 amino acid protein in the mouse [3]. The first 24 amino acids are highly conserved in various vertebrate species, while *PRCD* could not be identified in non-vertebrate species. They further found an approximately equal expression of canine *PRCD* in the RPE, photoreceptor, and ganglion cell layers. Heterologous expression experiments showed that wild-type *PRCD* is distributed diffusely throughout the cytoplasm, while mutant protein aggregated. Zangerl and coworkers proposed that *PRCD* has a critical role in the maintenance of rod photoreceptor structure, function, and/or survival; however, the gene’s function remains unknown [3].

To date, only two RP-causing mutations of the *PRCD* gene in humans have been reported. To date only two RP-causing mutations of the *PRCD* gene in humans have been reported. Zangerl et al. found a homozygous mutation in *PRCD* (c.5G>A p.C2Y) in a single human patient with autosomal recessive RP from Bangladesh [3]. The patient showed typical functional and morphological characteristics of the disease, such as attenuated arterioles, extensive bone spicule-like pigmentation, and pale deposits at the level of the RPE. Additionally, small patches of geographic atrophy near fixation in both eyes were observed [3].

Nevet et al. [5] examined 24 patients from an Israeli Muslim Arab village affected by RP and found a second pathogenic homozygous nonsense mutation (c.64C>T p.R22X) in *PRCD*. The relatively frequent existence of an autosomal recessive disease in an isolated population suggested a founder effect. The ophthalmologic evaluation confirmed the diagnosis of RP in all affected individuals. In addition to the characteristic morphological changes, signs of macular degeneration including macular edema, macular pucker, scarring and BEM were observed in the majority of patients.

We found a novel homozygous nonsense mutation in exon 1 of the *PRCD* gene in three siblings affected by RP: c.52C>T, p.R18X. The mutation produces a premature termination codon at amino acid position 18 of the PRCD polypeptide, possibly resulting in a truncated and non-functional protein. However, more likely, there is no such polypeptide as the transcript is predicted to undergo nonsense mediated decay. A second unknown heterozygous sequence variant was also observed in all affected siblings in exon 1 of *RBP3*: c.1694T>C, p.V565A. This missense mutation alters an evolutionary highly conserved valine residue into an alanine, and prediction programs consider this variant pathogenic (Mutation Taster). However, no other sequence variant in *RBP3* was identified, and consequently, the homozygous nonsense mutation in *PRCD* was considered causative for the autosomal recessive phenotype in this family, while the *RBP3* variant was thought not to be responsible for the disease. Neither variant has been reported in the literature, and is not present in any database (i.e., Exome Variant Server, dbSNP). However, the *RBP3* missense mutation could have a modifying effect on the RP phenotype.

Our patients presented with similar findings in the ophthalmological examinations as reported in previously described cases: myopia, posterior subcapsular cataract, bone spicule-like pigmentation, and attenuated arterioles were typical findings. All of our patients showed an advanced stage of disease. Interestingly, BEM due to patchy RPE atrophy was also present in all patients. The signs of macular degeneration were especially visible in the two female patients. This is in line with the morphological changes observed in the cases examined by Zangerl and Nevet [3,5]. BEM seems to be a hallmark of RP due to mutations in the *PRCD* gene.

BEM is a distinctive macular phenotype characterized by annular RPE atrophy with central sparing of the fovea. Initially, BEM was described in association with chloroquine retinopathy [11] but has been associated with various inherited retinal degenerations, also with RP [12-14]. However, in retinitis pigmentosa is not a common feature, and therefore, it is remarkable that nearly all patients with *PRCD* mutations reported in the literature carry this phenotypic characteristic.
